# Design and validation of a preparedness evaluation tool of pre-hospital emergency medical services for terrorist attacks: a mixed method study

**DOI:** 10.1186/s12873-022-00712-7

**Published:** 2022-09-03

**Authors:** Sadegh Miraki, Yasamin Molavi-Taleghani, Mohammadreza Amiresmaeili, Mahmood Nekoei-Moghadam, Hojjat Sheikhbardsiri

**Affiliations:** 1 Department of Medical Emergencies, School of Nursing and Midwifery, Shiraz University of Medical Sciences, Shiraz, Iran; 2 Health Management and Economics Research Center, Department of Health Services Management, School of Management and Medical Information Sciences, Isfahan University of Medical Sciences, Isfahan, Iran; 3 Health in Disasters and Emergencies Research Center, Institute for Futures Studies in Health, Kerman University of Medical Sciences, Kerman, Iran

**Keywords:** Disasters, Preparedness, Emergency Medical Services (EMS), Terrorist Attacks, Tools

## Abstract

**Introduction:**

Terrorist attacks are one of the human problems that affect many countries, leaving behind a huge toll of disabilities and deaths. The aim of this study was to use a mixed-method analysis to design and validate an evaluation tool for pre-hospital emergency medical services for terrorist attacks.

**Methods:**

The present study is a mixed-method (qualitative and quantitative) study that was conducted in two phases. In the qualitative phase (item generation), semi-structured interviews were conducted with 34 Iranian emergency medical technicians who were selected through a purposive sampling method and a scoping literature review was conducted to generate an item pool for the preparedness evaluation of Emergency Medical Services (EMS) in terrorist attacks. In the quantitative phase (item reduction), for validity of tool face, content and construct validity, were performed; for tool reliability, the test and retest and intra-class correlation coefficient were evaluated.

**Results:**

At the first stage, 7 main categories and 16 subcategories were extracted from the data, the main categories including “Policy and Planning”, “Education and Exercise “,“ Surge Capacity”, “Safety and Security”, “Command, Control and Coordination”, “Information and Communication Management “and “Response Operations Management”. The initial item pool included 160 items that were reduced to 110 after assessment of validity (face, content and construct). intra-class correlation coefficient (ICC = 0.71) examination and Pearson correlation test (r = 0.81) indicated that the tool was also reliable.

**Conclusion:**

The research findings provide a new perspective to understand the preparedness of pre-hospital emergency medical services for terrorist attacks. The existing 110-item tool can evaluate preparedness of pre-hospital emergency medical services for terrorist attacks through collecting data with appropriate validity and reliability.

## Introduction

Terrorist attacks are on the rise around the world, and so far, many countries have been affected by such incidents, and many people have been killed and injured due to these incidents [1]. Terrorist incidents are one of the most significant and dangerous man-made incidents that occur every year in our world, taking the lives of many people and frightening society [2]. Terrorism has many definitions and is used for a number of purposes, including the use of power, violence, or threats to obtain political goals through terror and harassment [3]. Terrorism is defined by the US Department of Justice and the Federal Bureau of Investigation as the illegal use of force or violence against persons or property in order to scare or intimidate the government, citizens, or any portion of it in order to advance political or social objectives [4]. A total of 158,520 terrorist operations and attacks have been carried out in the world from 1970 to 2018, of which Iraq has been the country with 10,000 terrorist attacks, Pakistan with 7200 cases, Afghanistan with 6600 cases, India with 3000 cases, and the Philippines with 2200 cases have the highest number, and Iran, despite being in the sensitive region of the Middle East, has 35 terrorist cases [5].

The disaster management cycle consists of four important phases: prevention & mitigation, preparedness, response, and recovery [6]. The preparedness phase is the activities and actions taken in advance to ensure an effective response to the adverse effects of hazards [7]. Also defined by the United Nations International Strategy for Disaster Reduction, preparedness includes the knowledge and skills that enable organizations, communities, families, and even individuals to effectively anticipate, respond to, and recover from the effects of disasters [8]. Disaster relief agencies and organizations must develop and practice response plans, as well as improve the necessary capabilities and capacities quantitatively and qualitatively [9].

Pre-hospital emergency as one of the important organs and organizations in such incidents that immediately enters the scene and serves the injured has a very important role in such incidents and is an important part of the public safety network that ensures the safety and health of citizens [10]. Iran has a long history of pre-hospital emergency medical care, dating back to 1975. The former ministry of health built up a system to address people’s requirements for emergency medical treatment when the roof of the Mehrabad airport collapsed, killing and injuring many people. As a result, the Tehran emergency center was constructed and opened, followed by the establishment of numerous other emergency facilities in other provinces. Emergency medical services (EMS) began using its equipment and facilities to provide emergency medical services in response to the effects of war and growing urban populations, and has made moves to expand the provision of these services across the country [11]. Pre-hospital emergency services in Iran are free and the contact number is 115 [12]. A pre-hospital emergency shift lasts 24 hours, during which two emergency medical technicians with a degree in emergency medicine, nursing, or anesthesia work and rest for 48 hours, and at least one technician is expected to work eight days of 24 hours per month [13].

The results of some studies showed that, unfortunately, the level of preparedness for dealing with these events is low and the medical emergency is less prepared to manage these events [14]. Studies by Annelie Holgersson et al. [15] and Westman Anton et al. [16] stated that differences in viewpoints on terrorism preparation and response among emergency responders were demonstrating the significance of empowering inter-organizational insights on safety culture, risk perception, and management practices, as well as understanding of the other organizations’ institutional logics and main tasks, in order to achieve an effective, collaborative response to terrorism-induced incidents.

The awareness and proper functioning of pre-hospital emergency staff and managers are important in order to save lives and serve the victims of these incidents [17]. Considering that the world’s countries have always been exposed to various military and terrorist threats from their enemies and given that today there are new changes, variations, and complexities in weapons of war, their use, and their effects, there is a need for different medical teams to increase their preparedness to manage such incidents [18]. Considering that there are various terrorist attacks in the world at the moment, and these incidents have caused a lot of financial and human losses to many innocent people in society, pre-hospital emergency has a very important role in saving lives and the health of people [19, 20]. One of the main obstacles to the accurate and scientific evaluation of the prehospital system preparedness in a terrorist incident is the lack of standard and holistic tools. Therefore, the aim of this study was to use a mixed-method analysis to design and validate a preparedness evaluation tool for pre-hospital emergency medical services for terrorist attacks.

## Methods

### Study design

The present study is a mixed-method (qualitative and quantitative) study that was conducted in two phases. In the qualitative phase for (item generation), semi-structured interviews were conducted with Iranian emergency medical technicians who were selected through a purposive sampling method, and a scoping literature review was done to generate an item pool for the preparedness evaluation of EMS in terrorist attacks. In the quantitative phase (item reduction), for validity of tool face, content and construct validity, were performed; for tool reliability, the test and retest and intra-class correlation coefficient were evaluated.

### Qualitative phase (Item generation)

To create the items, three major steps were taken: a) interviewing pre-hospital emergency experts using a descriptive phenomenology approach to identify and develop the concept of prehospital preparedness in terrorist incidents, b) scoping review, and c) incorporating steps 1 and 2 for item pool (Synthesis Research).

### Step 1: Conduct a Qualitative Study

This qualitative study was conducted in Iran, one of the most disaster-prone countries in the world. The study population included 34 pre-hospital emergency staff and managers who had practical experience of pre-hospital emergency system preparedness in a terrorist attack and had at least one experience of terrorist attacks. Participants were chosen using a purposeful sampling method with maximum diversity. Sampling was carried out through a semi-structured interview until data saturation occurred when the researchers concluded that further interviews would fail to provide new information. Participant experts included 9 pre-hospital managers, 23 pre-hospital emergency personnel, and 2 experts in the dispatch ward of the pre-hospital emergency center. Inclusion criteria for the interview All emergency medical experts who had a role or experience in terrorist attacks experts who had material to say (such as heads of emergency medical centers, assistants in the technical and operations department of emergency medical centers, experts in the emergency operations center, and experienced operational personnel across the country). Finally, these individuals were willing to participate in the study and interviews. Exclusion criteria for the interview: people who did not have expressive points and did not want to participate in the interview.

The interviewees answered a similar set of questions which began with “have you ever experienced the disaster preparedness exercises of the health system?”, “What terrorist incident have you been involved in so far and what was your role in that incident?”, “What problems and challenges have you had in managing a terrorist incident and what were the strengths and weaknesses of the incident?” The data collected in this stage was analyzed using a phenomenological methodological approach by Colaizzi’s method. The coding process is being managed using the trial version of the MAXQDA 16 Software. This study employed strategies recommended by Lincoln and Guba for data trustworthiness [20].

### Step 2: A Scoping Review

#### Search Strategy

We conducted a scoping review in 2020 to identify and evaluate the performance and level of preparedness of pre-hospital emergency teams in terrorist attacks around the world. For this purpose, we studied databases including ISI Web of Science, PubMed, Scopus, Science Direct, Ovid, Pro Quest, Wiley, and Google Scholar from January 1, 2000 to February 13, 2020. Using OR and AND, key words were combined and entered into database search boxes, including ((Terrorist incidents OR Terrorist accidents OR Terrorism attacks OR Terrorism attacks OR Terrorism OR Terrorists) AND (Violence OR Political OR conflict OR criminality OR war) AND (Prehospital emergency OR Prehospital emergency Care OR Emergency Health Service OR Emergency Medical Response OR First medical Responder OR Emergency MedicalAll synonyms of the key words were searched using MESH strategies.

#### Inclusion and exclusion criteria

The following studies were selected that met the conditions and criteria for inclusion in the study: Studies which were published in English, Studies which studied the performance of pre-hospital emergency medical services (EMSs) during a terrorist attack Studies which considered the use of force, violence, or threats to achieve political ends, studies which were published as originals or reviews. In addition, studies that meet the following exclusion criteria are excluded: studies whose full text was not accessible, Studies which described other areas of aiding during terrorist attacks, including anti-terrorist medical service rendered by military personnel, post-terrorist attack medical care rendered by the fire service, police, or boy scouts, or responses taking place in field hospitals, Studies which considered events including hacking into a government computer or cyber attacks on governmental websites, Studies which focused on other kinds of emergency responses, including Internet security, sanitation, and transportation, studies which were published as letters to the editor, commentary, case reports, case series, expert consensus, published national guidelines, or editorials.

#### Articles and Documents Selected

After selecting the desired studies, the requested information was collected and analyzed, and all steps were checked and performed according to the principles of the PRISMA checklist [21]. The first preliminary search was performed by two authors (SM and MA) separately; in the next step, independent reviewers (SM and MA) screened abstracts and titles for eligibility. When the reviewers felt that the abstract or title was potentially useful, full copies of the article were retrieved and considered for eligibility by both reviewers. If discrepancies occurred between reviewers, the reasons were identified and a final decision was made based on the third reviewer (HS).

### Step 3: combining steps 1 and 2 (Synthesis Research)

In this step, all components and characteristics obtained in the previous two steps were combined; redundant items were removed, and similar ones were merged. Independent of the scoping review and qualitative study, new sub-categories and categories were created. Since the new categories and subcategories served as the foundation for the pool of items, they were evaluated with greater sensitivity. The final table, containing the theme, category, subcategory, and codes, expands the main preparedness evaluation as converted into items. The research team examined questions and eliminated or modified several. Finally, an initial format for the preparedness evaluation tool of pre-hospital emergency medical services was prepared, consisting of 160 questions. Subsequently, the primary questionnaire’s validity was determined. The psychometric properties of the tool were examined for face, content, and construct validity, as well as reliability. For each item, a response scale was considered based on a 3-point Likert scale.

#### Quantitative phase (Item reduction)

##### **Face validity**

We used qualitative and quantitative face validity, qualitative and quantitative content validity, and structural validity to validate this tool. In determining the quality of face validity, 10 participants who were more expert were selected and semi-structured interviews were conducted face to face, and the ease of completion, legibility, grammar, and the writing style of items in terms of ambiguity, level of difficulty, and fitness were examined. In determining the quantity of face validity, 10 participants assigned a value to each item using the five-point Likert scale ranging from five (quite important) to one (not important at all). Frequency (%) Importance = Impact score the impact score was considered to be greater than 1.5 [22]. Participants of both qualitative and quantitative face validity were chosen using a purposeful sampling method [23].

##### Content validity

For qualitative content validity to be ensured, ten health professionals experienced in terrorist attack incidences were asked to express their corrective views in terms of grammar, the use of appropriate words, proper placement of items, proper scoring and appropriateness of the selected dimensions. The questionnaire items were revised in response to experts’ suggestions. Thus, most of the items were transcribed by adding, substituting more common and understandable words, which led to the clarification of the vague items. Content validity ratio (CVR) and content validity index (CVI) were used to evaluate the quantitative content validity. To begin, the CVR was calculated and a panel of experts was asked to rate each item on a three-point scale: necessary, useful, and not necessary. In this phase, the content validity ratio was calculated using the Lawshe formula (1975), which is acceptable with a score of 0.64 or greater [24]. CVR will be calculated using the following formula:

$$CVR=\frac{n_E-\frac{N}{\Upsilon}}{\frac{N}{\Upsilon}}$$

The criterion of “relevance” was used for each item on the one-point Likert scale to determine the content validity index. For this purpose, 10 experts were asked to determine the correlation between the questionnaire items and the subscales of the questionnaire on a Likert scale ranging from one (not relevant) to four (completely relevant). Finally, K* will be calculated as follows, using the agreement ratio for the relevance of each item (I-CVI) and the probability of the chance agreement. According to Polit, the minimum number of evaluators required to calculate kappa using this method is three; the number of evaluators will be 10 in the present study. Kappa values of 0.59–0.40, 0.74–0.60, and > 0.74 will be considered poor, good, and excellent, respectively. In this study, only items with kappa of at least 0.74 will be accepted [23].

$$\mathrm{CVI}=\frac{\mathrm{number}\kern0.17em \mathrm{of}\kern0.17em \mathrm{raters}\ \mathrm{giving}\;\mathrm{a}\;\mathrm{rater}\;3\;\mathrm{or}\;4}{\mathrm{total}\kern0.17em \mathrm{number}\kern0.17em \mathrm{of}\kern0.17em \mathrm{raters}}\kern0.5em {p}_c=\left[\frac{N!}{A!\left(N-A\right)!}\right]{.5}^N\kern0.5em {k}^{\ast }=\frac{\mathrm{I}\hbox{-} \mathrm{CVI}-{p}_c}{1-{p}_c}$$

##### **Construct validity**

Convergent validity, for human cognition, especially within sociology, psychology, and other behavioral sciences, refers to the degree to which two measures that theoretically should be related are in fact related. Convergent validity, along with discriminant validity, is a subtype of construct validity [25]. In general, tools are classified as reflective and formative. In reflective tools, the items that make up the dimensions of the tool are conceptually related and structural validity is required to examine the dimensions of the tool. In formative tools, the items that make up the dimensions of the tool are not conceptually related [26]. For formative tools structural validity cannot be done by factor analysis (exploratory-confirmatory) method because factor analysis requires a linear relationship between variables [27]. designed tool in this study was a checklist with nature of formative and we used convergent validity to perform the construct validity. Convergent validity, one aspect of construct validity, were examined using Spearman’s correlation. Accordingly, values ≥0.40 represented appropriate convergent validity [28] .

In determining the construct validity of this tool we used the Convergent validity and a similar tool with title “Developing of Iranian Pre-Hospital Emergency Preparedness Assessment tool in Emergencies and Disasters” [29] was sent to thirty of Emergency and Incident Management Centers n (MEAIMC) across the country and the centers were asked to rate this tool based on had to be completed (the level of readiness of the centers for each tool item: if the expected function was performed correctly and on time(2), if the expected function was performed, but its quality or timing was improper (1) and the expected function was not performed (0). Then, about a month later, the initial tools designed in this study were sent to the same thirty of MEAIMC and they were again asked to complete and send these tools. The collected data was entered into SPSS 22 and Spearman correlation coefficient test was used.

##### **Tool Reliability**

The test-retest method was used to implement the reliability of the instrument. For this purpose, the instrument was first provided to thirty MEAIMC independent of the construct validity stage and the necessary information was collected. Then, about a month later, the initial tools designed in this study were sent to the same 30 MEAIMC. the collected data was entered into SPSS 22 and Pearson correlation test was used. Ten MEAIMC independent of previous stages were chosen for this study to test instrument reliability. Two evaluators evaluated each MEAIMC independently. After the evaluators completed their evaluations of all MEAIMC, the collected data was entered into SPSS 22 and the reliability of the instrument was examined using Intraclass Correlation Coefficient (ICC).

## Results

### Qualitative phase (Item generation)

#### Step 1: Conduct a Qualitative Study

Table 1 shows how an original theme, pre-hospital emergency preparedness in terrorist attacks, was created, along with seven main categories and sixteen subcategories. The concept of pre-hospital emergency preparedness in terrorist attacks yielded 282 codes in the qualitative study.

**Table 1:** Categories and subcategories extracted from qualitative data

Main Categories	Subcategories
Policy and Planning	Design protocols and standard operations process
	Define responsibilities and duties
Education and Exercise	Increasing skills
	Holding maneuvers and practical trainings
	Culturing
Surge Capacity	Human resources
	Facility and equipment
Safety and Security	Protection
	Scene management
	Personal protection
Command, Control, Coordination	Unit Command
	Inter-organizational interaction (common language)
Information and communication management	Effective and integrated communication
	Data collection
Response Operation management	Triage and Evacuation
	Routing

#### Step 2: Scoping review

A scoping review was conducted in the first phase of the study, with the following results: The initial search yielded 794 documents using the specified search strategies. After duplicates, books, dissertations, and presentations were removed, the number of documents was reduced to 237. First, titles and abstracts were screened for those that were related to pre-hospital emergency preparedness in terrorist attacks, and 82 articles were found to be eligible. Then, after reviewing all 82 full-text papers, eight papers reported on pre-hospital emergency preparedness in terrorist attacks. 223 codes of pre-hospital emergency preparedness components in terrorist attacks were extracted from 8 related articles in a scoping review [28].

#### Step 3: combining steps 1 and 2 (Synthesis Research)

Following two meetings with the research team and experts, the third step involved removing duplicate items, merging similar ones, and finally reducing the number of items to 160 by selecting the most relevant ones, followed by the psychometric process.

### Results of the second phase of the study (face, content, and construct validity)

The quantitative phase of the study showed that 11 items were edited in qualitative determination of face validity and 149 items remained unchanged. In determining the quantitative face validity, 9 items should have been removed, but according to the method mentioned in this step, no item was removed and the tool was still considered with 160 items for the content validity stage. In a qualitative review of content validity, 22 items were modified, 4 were merged with other items, and 3 were transferred from one subgroup to another.156 items were considered for quantitative content validity. In order to evaluate the quantitative content validity, two indicators of content validity ratio (CVR) and content validity index (CVI) were used. In determining the content validity ratio, a total of 40 items were removed due to the content validity ratio score of less than 0.62. In determining the content validity index, 6 items were removed due to obtaining a K score of less than 0.74 (excellent), and thus the total number of tool items was fixed at 110. Then, based on the mean CVI scores of all items, the mean CVI of the whole tool was calculated, with 0.9 being the acceptable standard [30]. Table 2 shows that the 0.97 obtained in the present study is acceptable. Accordingly, the values of the Pearson correlation coefficient test (r = 0.72) showed appropriate convergent validity.

**Table 2:** Results of validity of measurements

Item	Impact score	CVR	CVI	K
1-A pre-hospital emergency preparedness plan or instruction for terrorist incidents has been developed.	5	0.8	1	1
2-A special plan to improve the level of preparedness and effective response to terrorist incidents has been pre-designed and developed.	**3.8**	**0.6**	**-**	**-**
3-Charts and processes related to terrorist incident response management have been periodically and regularly updated.	4.7	0.8	0.9	0.98
4-There is an annual risk assessment plan for terrorist incidents including risk identification, capacity and vulnerability.	4.9	1	1	1
5-The incident command system is defined and designed based on the national model of pre-hospital emergency in the incident field.	5	0.8	0.9	0.98
6-A standard program and process has been developed for how resources are distributed.	**3.8**	**0.4**	**-**	**-**
7-There are instructions for the initial assessment of the terrorist incident and the necessary resources for the implementation of the principles by the assessment team.	4.7	1	0.9	0.98
8-A plan has been designed and developed to follow up and fully monitor the provision of supplies and services required to carry out terrorist incident missions.	**3.8**	**0.6**	**-**	**-**
9-There are enough standard instructions for arranging resources, support equipment, and physical protection team members at the scene.	4.6	0.8	1	1
10-Lessons learned and experiences from previous incidents have been used in developing new plans and processes.	**3.8**	**0.6**	**-**	**-**
11-Necessary coordination has been made with experts to advise and support operations in response to various terrorist incidents.	4.2	0.8	1	1
12-There is an internal and external memorandum of understanding with agencies related to the management of terrorist incidents, including the military and security forces, to manage the response to terrorist incidents.	4.8	1	0.9	0.98
13-Special measures have been taken to replace key individuals in the incident command system chart.	**4.2**	**0.4**	**-**	**-**
14-There are instructions for leveling, activating and deactivating the response program to all types of terrorist incidents.	4.8	0.8	1	1
15-There are standard instructions for special response operations for a variety of terrorist incidents.	4.7	0.8	0.8	0.78
16-There are instructions for scoping review and recording of lessons learned, experiences, reports and actions taken related with terrorist incidents.	5	0.8	0.9	0.98
17-Description of the duties of the various pre-hospital emergency committees, including the determination of macro-preparedness policies, the adoption of the required instructions and standards related to the implementation of preparedness processes against terrorist incidents.	4.6	1	1	1
18-Responsible and substitute persons and their job descriptions in different positions of the incident command system as well as the standard response schedule of 0 to 2, 2 to 12 and more than 12 hours are specified and defined.	4.3	1	1	1
19-Pre-hospital emergency staff and operational personnel are aware of their job descriptions in accordance with the Terrorist Incident Response Operational Plan.	4.8	0.8	0.9	0.98
20-Necessary coordination has been made with the relevant authorities to hold self-defense training courses for managers and pre-hospital emergency staff.	4.9	1	0.9	0.98
21-The incident command system is implemented based on the roles and tasks and pre-determined people at the scene of the incident.	**4.9**	**1**	**0.6**	**0.64**
22-A continuing education program on how to use safety and personal protection equipment for operational personnel is tailored to the type of terrorist incident.	4.3	0.8	1	1
23-Specialized and experienced instructors in terrorist incidents are used to train and increase staff knowledge.	4.6	0.8	1	1
24-Senior pre-hospital emergency officials, especially the incident commander, have full knowledge of those in charge and their duties and role in the incident command system.	**3.8**	**0.4**	**-**	**-**
25-Dedicated training courses are held annually for dispatch and operations management staff on how to run and guide ambulances continuously and coordination between hospitals.	5	0.8	1	1
26-Authentic books and resources related to terrorist incidents are available at pre-hospital emergency bases for the use and study of shift staff.	4.2	0.8	0.9	0.98
27-Necessary measures have been taken to distribute the duties fairly and correctly and not to entrust multiple responsibilities and duties to one person.	**3.8**	**0.4**	**-**	**-**
28-There is a scoping monitoring and evaluation of the educational-skills status of pre-hospital emergency personnel in terrorist incidents.	3.8	0.8	1	1
29 Training of all staff and operational staff on their roles, responsibilities and job descriptions in terrorist incidents is carried out periodically.	4.3	0.8	1	1
30-The content of the personnel training program is developed and implemented in accordance with the model published by the Ministry of Health.	**3.8**	**0.6**	**-**	**-**
31-Training of all staff and operational staff on their roles, responsibilities and job descriptions in terrorist incidents is carried out periodically.	4.8	0.8	1	1
32-The effectiveness of the training given to pre-hospital emergency staff in relation to terrorist incidents is evaluated periodically.	4.9	1	1	1
33-The Disaster Management and Terrorism Management Matrix is designed to identify what categories of employees should be trained for what subject.	**3.8**	**0.4**	**-**	**-**
34-Topics related to the management of terrorist incident operations are included in the training topics of the Training Office of the pre-hospital emergency Center.	4.7	0.8	0.8	0.78
35-A memorandum of understanding has been established to use and employ qualified instructors of the security and law enforcement agencies to teach the security points of terrorist incidents to pre-hospital emergency staff.	4.6	0.8	0.49	0.78
36-Necessary trainings have been given to the managers and emergency operations staff regarding the knowledge and how to use the correct and principled means of communication, urban planning and map reading.		0.8	0.9	0.98
37-A local memorandum of understanding has been signed between the pre-hospital emergency department and the Radio and Television to create a culture and increase the level of public awareness about providing first aid to the injured and victims of these incidents before the emergency arrives.		1	0.8	0.98
38-Personnel have been trained in the communication protocols provided.		**0.4**	**-**	**-**
39-Appropriate measures have been devised to motivate and interest pre-hospital emergency staff to participate in training related to terrorist incidents and increase their awareness.		1	1	1
40-Training needs assessment and setting a minimum standard of adequacy of staff performance to deal with terrorist incidents.		1	1	1
41-Children and adolescents receive the necessary training to deal with terrorist incidents while in school.		**0.6**	**-**	**-**
42-Orientation sessions are held regularly and periodically at least once a month in each shift for staff at the bases.		**0.6**	**-**	**-**
43-The passive defense of the pre-hospital emergency center holds the necessary training courses to increase the awareness of all staff on a periodic basis.		0.8	1	1
44-Periodic (at least annual) operational exercises are planned and carried out based on possible scenarios of terrorist incidents at different operational levels.		1	1	1
45-Monitoring and evaluating the effectiveness of exercises, identifying strengths and weaknesses, and reviewing terrorist response programs.		0.8	0.49	0.98
46-Necessary measures have been taken regarding the initial acquaintance of the pre-hospital emergency personnel with the common language, dialects and culture of the people in the area where they are taking shifts.		**0.4**	**-**	**-**
47-Practical defensive driving training courses are held to increase the skill level and readiness of pre-hospital emergency staff.		0.8	1	1
48-Common exercises will be held with other organs involved in responding to terrorist incidents to achieve greater coordination in incident scene management.		1	1	1
49-Pre-hospital emergency staff have the necessary physical and mental readiness to participate in the exercise, including motivation, seriousness and desire.		0.8	1	1
50-Training and exercise programs have been developed and implemented to improve stage management skills for staff.		1	1	1
51-Physical fitness courses for pre-hospital emergency staff and managers are held periodically and regularly.		0.8	1	1
52-The contact number list of all managers and operational staff, especially employees who shift in important and vital places, is available and is updated regularly and periodically.	4.6	0.8	1	1
53-All lessons learned from events and exercises are scopingally recorded by a specific person.	**3.8**	**0.6**	**-**	**-**
54-The physical fitness of pre-hospital emergency personnel and managers is monitored periodically and regularly.	**3.8**	**0.6**	**-**	**-**
55-There are necessary measures for continuous monitoring of physical health and providing medical services to pre-hospital emergency staff during terrorist incidents.	4.9	1	1	1
56-Details of the response to terrorist incidents are specified as special incidents in the incident command system.	**3.8**	**0.6**	**-**	**-**
57-In critical and important centers, based on the capacity and capability of the emergency medical center of that province and the opinion of relevant experts, a special operations team has been considered to respond to terrorist incidents.	4.7	0.8	0.6	0.98
58-Pre-hospital emergency staff are actively involved in designing and reviewing programs related to terrorist incidents.	4.9	0.8	0.9	0.98
59-A memorandum of understanding has been reached with insurance organizations to insure managers and operational personnel in the event of possible damage.	**3.8**	**0.4**	**-**	**-**
60-There are appropriate instructions for handling and following up on the affairs of injured operational staff, including incident insurance.	4.6	0.8	1	1
61-Necessary measures have been devised to track the personnel on duty.	**3.8**	**0.2**	**-**	**-**
62-Strategies for caring for employee families are designed to increase employee flexibility to recall and extend longer working hours.	4.7	1	1	1
63-Relevant pre-hospital emergency authorities have developed a program to identify potential manpower capacities.	**3.8**	**0.6**	**-**	**-**
64-The correct arrangement of more experienced and skilled operational staff in shifts is emphasized by importance of the presence of an experienced technician with a new technician.	4.6	0.8	1	1
65-There is a process for recruiting, distributing and properly employing pre-hospital emergency staff, including determining the appropriate rotation shift, determining the appropriate force mix for units, especially operational units.	4.7	0.8	0.9	0.98
66-Necessary measures have been considered to complete the form and follow up on personnel injury reports.	**3.8**	**0.6**	**-**	**-**
67-A process is envisaged to honor the services of operational personnel involved in responding to terrorist incidents and to appreciate them and their families.	4.3	1	1	1
68-In order to reduce the medical errors of the operational staff, a plan has been developed and operated for periodic rotation and change of the staff shift.	4.6	0.8	1	1
69-Necessary measures have been considered regarding psychological support and mental health of employees and their families in the short and long term.	5	1	1	1
70-In each shift, specialized and trained human resources are allocated on a case-by-case basis to prepare for terrorist incidents.	4.8	0.8	1	1
71-Continuous supply, distribution and monitoring of medical and non-medical equipment required for urban and road pre-hospital emergency ambulances in accordance with various terrorist incidents.	4.1	0.8	1	1
72-The free time of the operational personnel in the shift is used optimally and usefully in order to prepare and increase their skill level.	**3.8**	**0.6**	**-**	**-**
73-Pre-hospital emergency equipment is updated and completed according to the latest standards, and all ambulances have the same arrangement.	4.4	0.8	1	1
74-The geographical map of the covered area and the main and secondary traffic routes of pre-hospital ambulances are drawn and available at the emergency operations center.	4.8	1	1	1
75-Necessary measures have been taken to provide and equip ambulances for terrorist attacks, depending on the type of incident.	4.9	1	1	1
76-The plan is to provide alternative equipment to respond to terrorist incidents.	3.8	0.8	0.6	0.58
77-Equipment and support resources for responding to terrorist incidents are available for at least 72 hours in the store of the Emergency Operations Center.	4.8	1	1	1
78-Necessary measures have been taken in connection with concluding contracts with pharmaceutical and medical equipment companies to supply drugs and equipment required in terrorist incidents.	5	1	1	1
79-All emergency equipment and devices have property numbers to prevent them from disappearing at the scene of the incident.	**3.8**	**0.4**	**-**	**-**
80-A specific list of all the equipment and supplies needed for various types of terrorist attacks has been prepared.	4.7	1	0.49	0.78
81-All types of replacement ambulances with all equipment are envisaged to support the response to terrorist incidents at 20% of the original capacity.	4.4	0.8	1	1
82-A memorandum of understanding has been reached with the rescue and security forces to be present in time to provide security at the scene of the incident for pre-hospital emergency staff.	4.8	1	0.9	0.98
83-All members of the terrorist incident management system are present at the scene with a special cover and label and can be identified.	4.2	0.8	0.9	0.98
84-Necessary measures have been taken to restrict the access of ordinary people to the ambulances present at the scene of the incident and to enter the front or rear cabins of the ambulances.	4.7	1	1	1
85-There are good guidelines for the correct location of a pre-hospital emergency ambulance in terms of proper geographical distribution, security, and facilitation of physical access to the scene.	5	1	0.9	0.98
86-There are instructions required to inspect the scene of the incident for hazardous materials and conditions by the Chief of Safety and Security of the Command System.	5	1	1	1
87-In order to manage various terrorist incidents, the necessary coordination has been done with partner and support organizations.	4.1	0.8	1	1
88-Necessary coordination has been done with organizations related to incident management, including military, security, fire, etc., to zoning the scene of the incident during the terrorist attacks.	4.3	0.8	1	1
89-There is a valid way to identify pre-hospital emergency staff and patients and ordinary people present at the scene.	**3.8**	**0.6**	**-**	**-**
90-The necessary instructions have been devised to maintain the equipment of the injured who are not conscious and to deliver them to the relevant authorities.	4.6	0.8	0.9	0.98
91-There is an instruction or process for identifying hazardous materials and decontamination at the scene by pre-hospital emergency staff.	4.4	0.8	1	1
92-An appropriate mechanism has been considered for combining pre-hospital emergency laws and disciplinary laws to carry out security operations at the scene of an incident.	**3.8**	**0.4**	**-**	**-**
93-Personal protective equipment suitable for all types of terrorist incidents is provided for pre-hospital emergency staff to be present at the scene.	4.9	1	1	1
94-Pre-hospital emergency operations personnel are fully aware of the proper and principled use of personal protective equipment in terrorist incidents.	4.2	0.8	0.9	0.98
95-Necessary coordination has been made with security teams to move and transfer the corpse.	**3.8**	**0.6**	**-**	**-**
96-The pre-hospital staff quarantine protocol is in place to prevent the spread of contaminated and hazardous materials from biological terrorist incidents.	4.8	0.8	0.8	0.98
97-There is a proper way to identify pre-hospital emergency personnel and identify them when they enter the scene of a terrorist incident.	**3.8**	**0.4**	**-**	**-**
98-There is a standard decontamination process for patients, staff, equipment and corpses in biological terrorist incidents.	5	0.8	1	1
99-Necessary measures have been considered to inspect the suspected abandoned vehicles at and around the scene of the incident and to evacuate them to safe places.	**3.8**	**0.6**	**-**	**-**
100-There is a specific protocol to prevent ordinary people from entering the scene of the incident and creating congestion and interference in the work of emergency personnel.	**3.8**	**0.4**	**-**	**-**
101-There are instructions for the safe pre-hospital transfer process for the injured or sick person contaminated with hazardous materials from terrorist incidents.	4.6	0.8	1	1
102-The incident commander has the necessary scientific, physical and psychological capabilities to manage and command terrorist incidents.	4.3	0.8	1	1
103-To manage the response, the unit command is specified according to the level and type of terrorist incident in the incident area.	4.9	1	1	1
104-Necessary measures have been considered in relation to the vaccination of personnel in the face of diseases and respiratory problems.	**3.8**	**0.6**	**-**	**-**
105-Necessary measures have been considered to control all personnel present in the terrorist incident regarding the occurrence of special symptoms that result from the incident.	**3.8**	**0.8**	**0.8**	**0.64**
106-The pre-hospital incident command system is designed and implemented for all types of terrorist incidents.	4.9	1	1	1
107-The incident command post is set up at the scene of the incident in accordance with the chronological order of events and the operational plan of the incident specified in the operations guidance center, and can be easily identified.	4.1	0.8	0.8	0.88
108-Numerous periodic meetings (at least once a year) have been held between the directors of various organizations involved in terrorist incidents to better understand their duties and responsibilities, to establish interaction and to update the required actions.	4.4	0.8	0.9	0.98
109-A memorandum of understanding has been drafted with the protection of important and vital places in the country to confirm the identity and facilitate the rapid entry and exit of pre-hospital emergency operations staff at the time of response.	5	0.8	0.9	0.98
110-The role of partner and supporting organizations in responding to terrorist incidents is at the pre-hospital operations guidance center for further coordination.	3.9	0.8	1	1
111-Memoranda of Understanding have been concluded and are available with private, military and charitable hospitals near critical areas for the reception of the injured or sick.	4.9	1	1	1
112-Necessary measures have been devised to obey all managers and operational personnel of the main commander of the scene.	**3.8**	**0.6**	**-**	**-**
113-There is a plan or instruction to use the helicopter in terrorist attacks and to obtain flight permits quickly with the relevant organizations.	4.5	1	1	1
114-Necessary coordination has been made with responsible partner and support organizations outside the geographical area in order to respond to terrorist incidents.	4.1	0.8	1	1
115-A memorandum of understanding has been concluded with partner, support and private sector organizations to provide the necessary resources and equipment in the event of a terrorist incident.	4.9	0.8	1	1
116-Coordination with local military and security organizations to ensure the safety of staff, ambulances on the scene, casualties, equipment and pre-hospital emergency resources.	4.7	0.8	0.9	0.98
117-Numerous meetings have been held between emergency security and security of vital places to confirm the identity and rapid entry and exit of operational personnel to these places in critical situations with the coordination of competent structures at the provincial and city levels and the security of the relevant University of Medical Sciences.	**3.8**	**0.4**	**-**	**-**
118-A comprehensive database including telephone numbers, contact methods and addresses of all members of the organization has been prepared to participate in responding to terrorist incidents.	3.6	0.8	1	1
119-An appropriate communication program with a variety of efficient and multi-layered communication platforms between different levels of people present at the scene of the incident has been designed in the operations guidance center of the organization.	5	1	1	1
120-The necessary equipment for communication, including radio or telecommunication communication between the internal and external units of the participating organization in response to terrorist incidents has been provided.	4.8	0.8	1	1
121-Necessary arrangements have been made with the relevant authorities to take off and use the helicopter in these incidents and to obtain flight permits quickly.	**3.8**	**1**	**0.6**	**0.58**
122-Updated form Contact information for other centers and organizations related to responding to terrorist incidents is available at the organization's operations guidance center.	5	1	1	1
123-Necessary public information has been provided through the local media about the aims, process and location of the incident to prevent intimidation, panic, rumors and disturbance of public order.	5	1	1	1
124-There is a standard instruction or action plan for the process of gathering information about a terrorist incident from the time the incident is confirmed to the end of the incident.	5	1	1	1
125-There is a program to activate the rapid alert system and determine the level of alert to internal and external organizational units and operational response groups.	4.4	0.8	1	1
126-An instruction or program has been designed and developed to inform the danger and to produce and transmit clear messages from the incident situation to the users of the messages, including employees, relevant organizations and people.	4.8	0.8	1	1
127-Appropriate information systems inside and outside the organization in terrorist incidents have been designed and developed.	4.1	0.8	0.9	0.98
128-There are instructions for creating a local database on terrorist incidents and documenting all incident information.	3.9	0.8	0.9	0.98
129-A trusted liaison and spokesperson in the field of health has been identified to communicate with the public, the media and health officials regarding the various stages of responding to a terrorist incident.	4.2	1	1	1
130-A multi-layered communication program and appropriate communication platforms between operational units have been prepared and designed.	**4.6**	**0.4**	**-**	**-**
131-Planning has been done to record the information and identify the injured.	4.6	0.8	1	1
132-Necessary measures have been considered to ensure the security of information on the terrorist incident based on the availability, confidentiality and integrity of the information.	4.8	0.8	1	1
133-Necessary measures have been taken to complete and update the information related to the incident, the injured and the dead quickly and continuously.	**4.6**	**0.6**	**-**	**-**
134-There are clear instructions and procedures for triage of victims of terrorist incidents at the scene.	5	0.8	1	1
135-A plan has been developed and implemented to obtain information from reliable sources.	**4.6**	**0.6**	**-**	**-**
136-The equipment and supplies needed to triage the victims of terrorist incidents are available in accordance with the geographical area.	5	1	1	1
137-There is a suitable training program for the triage team, based on the standard triage of the victims of terrorist incidents.	4.8	0.8	0.9	0.98
138-The local standard protocol and process for evacuating casualties from terrorist incidents is available at the Medical Emergency and Incident Management Center.	4.9	1	1	1
139-Planning has been done in order to acquaint the pre-hospital emergency staff with various methods of horizontal evacuation, vertical evacuation and complete evacuation.	5	1	1	1
140-Instructions or the process of selecting evacuation models (geographic model, resource model, and patient status model) for various types of terrorist incidents are available at the Incident and Emergency Management Center.	4.6	0.8	1	1
141-A list of approved and reputable mass media has been prepared in advance to inform the news and ways to communicate with them.	**4.6**	**0.4**	**-**	**-**
142-Instructions for familiarizing pre-hospital emergency staff with a safe emergency evacuation plan, exit routes for evacuating public, critical and important local facilities.	5	1	1	1
143-Necessary coordination has been done with hospitals close to important and vital places in relation to the rapid admission of patients to terrorist incidents and proper cooperation with pre-hospital emergency ambulances.	**4.6**	**0.4**	**-**	**-**
144-Danger maps of important and vital local places have been prepared by the University Operations Guidance Center.	5	1	1	1
145-An experienced person or persons are assigned to oversee all stages of the triage.	**4.6**	**0.8**	**0.6**	**0.58**
146-There is a specific plan for determining the routes of entry and exit of ambulances and pre-hospital emergency staff in terrorist incidents.	4.9	1	1	1
147-Geographic satellite imagery (live, recorded) of the local community is available at the pre-hospital emergency center dispatch unit.	4.7	1	1	1
148-Specific planning has been done to form a special team in the Emergency Medical Management Center.	**4.6**	**0.6**	**-**	**-**
149-Appropriate mechanism for creating a pad (helicopter landing point) is provided due to the dynamic scene of the incident.	3.9	0.8	0.9	0.98
150-An appropriate mechanism has been envisaged to establish a temporary morgue to better manage the bodies and the dead.	**4.6**	**0.4**	**-**	**-**
151-Necessary coordination has been made with the police and the corridors to open the routes for ambulance traffic and to create shortcuts and alternative routes.	4.8	0.8	0.9	0.98
152-Regular visits of pre-hospital emergency managers to important and vital places and coordination with the officials of these places to determine and review the entry, exit and alternative routes.	4.2	1	0.6	0.78
153-Necessary coordination has been made by security and military teams to ensure the security of emergency personnel, ambulances and their equipment.	**4.6**	**0.4**	**-**	**-**
154-Necessary measures have been considered regarding the signboards in case of emergency and the route in terrorist incidents.	**4.6**	**0.6**	**-**	**-**
155-There is a guide program for choosing the right route to lead the pre-hospital emergency fleet in the context of Internet and traditional methods in the dispatch of the pre-hospital emergency operations center.	4.9	1	0.9	0.98
156-Patrols and urban planning, especially in the streets and important areas, have been carried out by emergency operations personnel and dispatch personnel.	**4.6**	**0.8**	**0.6**	**0.6**
			S-CVI /Average=0.97	

### Tool reliability results in

Intra-class correlation coefficient (ICC = 0.71) examination and Pearson correlation test (r = 0.81) indicated that the tool was also reliable.

### The final tool

The checklist for evaluation of the preparedness of pre-hospital emergency medical services for terrorist attacks includes 110 items divided into four dimensions of “Policy and Planning”, “Education and Exercise”, “Surge Capacity”, “Safety and Security”, “Command, Control and Coordination”, “Information and Communication Management” and “Response Operations Management”. The checklist items were weighed and scored based on the opinions of the experts experienced in disaster response in Iran. The checklists are scored based on the functions that are expected to be performed in the preparedness stage.

Mark No. 2 if the expected function was performed correctly and on time.

Mark No.1 if the expected function was performed, but its quality or timing was improper.

Mark No. 0: The expected function was not performed.

### The cutoff point of the tool

According to the total scores of 50 items on the Likert three-point scale, the maximum and minimum scores were 100 and 0, respectively. In this tool, scores of 0-33.5 indicate poor preparedness, scores of 33.6-66.5 indicate moderate preparedness, and scores of 66.6-100 indicate good preparedness. In addition, by using the linear transformation formula, converting the score obtained from the tool into a percentage and comparing it with the maximum and minimum scores of the tool, the level of preparedness of each health sector is calculated and interpreted in terms of percentage. 0-33.5% show poor preparedness, 33.5-66.5% show medium preparedness, and 66.5-100% show good preparedness.

## Discussion

Unfortunately, many terrorist incidents happen in different countries every year with many innocent victims. Terrorist attacks are made with other intentions, and the agent is seeking to obtain special privileges [31]. Education and Exercise, Surge Capacity, Safety and Security, Command, Control and Coordination, Information and Communication Management, and Response Operations Management are the main components affecting the pre-hospital emergency preparedness for terrorist attacks.

According to an analysis and assessment of published studies, different countries, both developed and developing, have a fresh and distinct approach to these occurrences and are attempting to improve their preparedness and capacities in many domains to manage and tackle these incidents [31]. Despite the fact that varied levels of preparedness have been investigated and claimed in different countries, these studies reveal low levels of preparedness in various countries and communities [32]. According to DiMaggio et al., healthcare providers are less likely to respond to terrorism-incidents like smallpox epidemics, terrorist chemical incidents, and nuclear bombs, but they are enthusiastically ready for natural disasters [33]. A regular training plan that allows individuals involved in emergency situations to exercise their roles and responsibilities before real disasters occur [33]. Individuals are not only prepared for their roles and tasks through exercise, but they are also able to identify planning flaws [34].

According to the results of this study, Policy and planning were found to be some of the foundations for pre-hospital emergency medical services preparedness for terrorist attacks. This results is consistent with findings of the study of Bart Schurman et al. [35] Who have introduced several key factors for the management of terrorist incidents, one of which is planning, and recommended to the pre-hospital emergency organization’s planners and managers that they have previous experience in designing special programs in this field to use. But in a study by Chartoff et al. [36] the discussion of planning has been challenged, and the existence of previous plans and protocols in an organization for a particular event or action requires experienced and experienced planners who have several times Participate in those specific operations and have an active role, and these planners should avoid existing political and managerial attitudes and do not include personal issues and job privileges in these plans.

education and exercise, which includes three subcategories; Increase knowledge, increasing awareness and increase skills and practice. Elena A. Skryabina et al. [37] in their study, showed that personnel who had already received training were more prepared to deal with terrorist incidents than personnel who had not been trained, and this shows the important role of training in managing terrorist incidents, also Tanya Jean Hockett et al .[38], by analyzing and categorizing a series of open-ended responses, determined that the most important benefit of exercise and practice participation is understanding plans, and enhancing communications. However, in a study conducted by Beck et al. [39] this finding is not very consistent and in their study, they pointed out that performing maneuvers is not very effective in training exercises because employees and executives, given the knowledge that this Such training and exercises are unrealistic, they do not cooperate very seriously.

Another main category mentioned by the participants was surge capacity, which plays an important role in terrorist attacks and is directly related to saving more lives. In this regard, the findings of Weifeng Shen’s [40] study show that four elements are effective for success and better management of important events and disasters, and the surge capacity of one of these elements.

Safety and security were also one of the main categories extracted from the analysis of interview information with pre-hospital emergency experts. Pre-hospital emergency personnel and managers involved in terrorist incidents must be aware of safety and security issues in order to save both their lives and the lives of those injured in these incidents. In this regard, Liam Fan [41] and Rob I Mobi [42] state that the security of critical incidents and terrorist attacks is possible with the cooperation and interaction of relevant organizations such as the police and security forces, and the police should be able to recruit health care personnel. Protect at the scene. However, in the study conducted by Cvetkovic et al. [43], it was stated that the security and safety of the scene is outside the pre-hospital emergency duties and the necessary coordination and memoranda of understanding must have been done with the security teams and the police in order to save the lives of the emergency staff.

Command, control and coordination was another main category extracted from interviews with participants in this study. All organizations and organs involved in terrorist incidents must convene in advance to coordinate and interact with each other and coordinate on the ground. In this regard, Framert et al. [44] emphasize that coordination and interaction between organizations involved in the management of terrorist incidents is very important, and this affects patient management, facilities and resources, patient transfer triage, and other factors. In his study, Ardalan [45] stated that coordination is always a challenge in accident management and the health system is required to use mechanisms for better cooperation between partner organizations and those responsible for the accident so that information can be exchanged effectively between organizations and cause more coordination in incident response management.

Information and communication management is also one of the important issues to improve the readiness of the pre-hospital emergency organization in the management of terrorist incidents, which was mentioned by the interviewees. Today, with the advancement of technology, different ways and means of communication have been created that the pre-hospital emergency system must choose the best and most efficient way of communication in advance in order to be able to communicate effectively and continuously with each other in different scenes. In this regard, Rogers et al. [46] in their study, the importance of effective communication in reducing disease and mortality in the event of a terrorist attack, and they also stressed that the choice of effective means of communication should be made by relevant professionals. And select an appropriate method that has the least defects and has been tested and evaluated several times so that pre-hospital emergency staff and managers can communicate well with each other at the scene of a terrorist attack.

The response operation was the last category of the main categories. In discussing the response to terrorist attacks, the correct and principled triage of the wounded and victims plays an important role in reducing the number of casualties in these incidents. In this regard, the findings of a study conducted by Pepper et al. [47] showed that triage of injured and victims of terrorist incidents has its own problems and complexities that pre-hospital emergency officials should be fully aware of these cases and be able to Do a good triage of the injured. However, in a study conducted by William et al. [48], they argued differently that the injured and victims of terrorist attacks should be treated differently from other casualties, and that all casualties should be treated in a specifically hospital with specific measures transferred.

The tools developed in this study were designed and developed as a result of interviews with experts in the field of pre-hospital emergency and using their experiences in preparing for and managing terrorist attacks and reviewing existing information texts and evaluation tools. In 2020, Beyrami et al. [49] designed and validated pre-hospital emergency preparedness assessment tools for accidents and disasters. In the construct validity section, the tool was sent to 30 pre-hospital medical emergency and accident management centers across the country, and the centers were asked to complete their readiness according to the tool items. The main difference between the tools of the present study and the above tools was that the above tools measured the level of pre-hospital emergency preparedness in accidents and disasters, whereas we measured the level of pre-hospital emergency preparedness in terrorist attacks. In 2015, Heidranloo et al. [50] The researchers of this study designed and validated the hospital performance appraisal tool in the face of natural disasters. The retest was measured in 50 hospitals in the country; the researcher of this study performed and used qualitative face validity with the participation of 15 experts in the field of health in disasters and disasters. As an evaluator, they performed the qualitative and quantitative content validity stages with 15 accident and disaster experts, and the method of comparing known groups or differential validity was used to perform construct validity. An important difference between this tool and the tool designed in our study is that the above tool is used to assess the operational readiness of hospitals, whereas our tool is specifically designed to evaluate the pre-hospital emergency preparedness functions in terrorist attacks. Sheikh Bardsiri et al. [51] In 2016, they conducted a study entitled “Assessing the readiness of the health department of the medical schools in the southeastern Arctic of Iran against earthquakes through the implementation of a full-fledged operational exercise.” The exercise evaluation tool included a checklist for evaluating the managerial performance of the healthcare department, which was prepared by the exercise design team. This checklist had 13 functional dimensions and contained 72 items. Comparing the tools used in the above study with the tools designed in the present study, it can be concluded that the main weakness of the checklist used in the previous tools was that this checklist focused only on the management functions of the university health department and other functional aspects of disaster management. Pre-hospital and hospital emergency operations; management of communicable and non-communicable diseases; environmental health, nutrition, psychosocial support, drug response operations, laboratory services, etc. were ignored, and on the other hand, the checklist was prepared in terms of validation and has only content validity but lacks reliability. Tang et al. [52], in 2014, Through a scoping review of available preparation tools, they conducted a study entitled “Building an Assessment Tool for Hospital Emergency Preparedness in China.” In this study, after a scoping review, they presented a tool for evaluating hospital exercises. The instrument designed in this study has 68 items that examine the vulnerability of the hospital in the fields of structural and non-structural and, out of 68 items, 21 items are open-ended questions. Examining the instrument revealed that there is no item to examine the performance dimensions of the hospital, and even in the non-structural area, it only deals with a few dimensions of this area, while today the expectation from the readiness assessment tools is to be able to use the capacity of hospitals. and assess the level of safety required to provide services by hospital staff, in addition to the fact that detailed information on the validity and reliability of the instruments produced in the study was not provided. The researcher himself had introduced the main limitation of the studies selected in the final analysis to make the tools: the lack of validity and reliability of the tools.

### Limitations

#### Limitations of the qualitative section

- Lack of access to some experts and specialists in the field of pre-hospital emergency in order to interview these people and gain their experiences and opinions.
- Lack of access to some information about terrorist attacks in our country due to the confidentiality and security of this information.
- Scattering of people to be interviewed in different cities and provinces of the country.
- Reluctance of some pre-hospital emergency staff to participate in terrorist attacks to conduct interviews.
- Fear and anxiety of some pre-hospital emergency staff about expressing some weaknesses observed in the terrorist incident in question.

#### Limitations of quantitative section

Lack of access to some professors and experts in the field of pre-hospital emergency in order to complete the forms of different psychometric stages of the primary study tool.

The unwillingness of some knowledgeable professors to cooperate and participate in the study.

Long waits and delays in order to receive answers from some professors and experts to complete the evaluation forms of different stages of psychometrics of the primary study tool.

Lack of cooperation of some pre-hospital emergency medical centers in the country in completing the designed basic tools.

Lack of accurate and principled completion of basic tools designed by some pre-hospital emergency medical centers in the country.

## Conclusion

Given that Iran is located in a sensitive and accident-prone region of the Middle East and that, in recent years, unfortunately, several terrorist attacks have occurred in different provinces of this country, all organizations involved in such incidents, especially pre-hospital emergencies, should increase their readiness to better manage such incidents. The studies carried out in the initial design of this tool showed that, unfortunately, most of the accident and medical emergency management centers in the country are not reasonably prepared to manage and respond to terrorist attacks and must improve their level of preparedness for this. This developed tool by our team can play an essential role in increasing the level of readiness and operational capacity of these centers against terrorist attacks by removing the existing obstacles and challenges and evaluating the standard and accurate level of readiness of medical and emergency management centers in the country.

Pre-hospital emergency preparedness in order to properly manage and respond to terrorist attacks requires the development and further training of response programs and quantitative and qualitative upgrades of the necessary capabilities and capacities, which require the establishment and upgrading of specific preparedness functions in the pre-emergency response operational plan. The vacuum created by a standard and comprehensive tool for assessing pre-hospital emergency preparedness in terrorist attacks is one of the main obstacles to an accurate and scientific evaluation of this readiness. The existence of a standard tool for measuring and evaluating the level of pre-hospital emergency preparedness in terrorist attacks is very important and important, and pre-hospital emergency teams should periodically assess their level of readiness based on this tool and evaluate it.

## Data Availability

The datasets generated and/or analyzed during the current study are not publicly available due to restrictions of the Ethics Committee of Kerman University of Medical Sciences. For available data, please contact: kmu_Research@yahoo.com.
